# Disruption of neonatal cardiomyocyte physiology following exposure to bisphenol-a

**DOI:** 10.1038/s41598-018-25719-8

**Published:** 2018-05-09

**Authors:** Manelle Ramadan, Meredith Sherman, Rafael Jaimes, Ashika Chaluvadi, Luther Swift, Nikki Gillum Posnack

**Affiliations:** 1 Sheikh Zayed Institute for Pediatric and Surgical Innovation, Children’s National Health System, Washington, USA; 2 Children’s National Heart Institute, Children’s National Health System, Washington, USA; 3 Department of Pediatrics, Department of Pharmacology & Physiology, School of Medicine and Health Sciences, George Washington University, Washington, USA

## Abstract

Bisphenol chemicals are commonly used in the manufacturing of polycarbonate plastics, polyvinyl chloride plastics, resins, and thermal printing applications. Humans are inadvertently exposed to bisphenols through contact with consumer products and/or medical devices. Recent reports have shown a link between bisphenol-a (BPA) exposure and adverse cardiovascular outcomes; although these studies have been limited to adult subjects and models. Since cardiac physiology differs significantly between the developing and adult heart, we aimed to assess the impact of BPA exposure on cardiac function, using a neonatal cardiomyocyte model. Neonatal rat ventricular myocytes were monitored to assess cell viability, spontaneous beating rate, beat rate variability, and calcium-handling parameters in the presence of control or bisphenol-supplemented media. A range of doses were tested to mimic environmental exposure (10^−9^–10^−8^M), maximum clinical exposure (10^−5^M), and supraphysiological exposure levels (10^−4^M). Acute BPA exposure altered cardiomyocyte functionality, resulting in a slowed spontaneous beating rate and increased beat rate variability. BPA exposure also impaired intracellular calcium handling, resulting in diminished calcium transient amplitudes, prolonged calcium transient upstroke and duration time. Alterations in calcium handling also increased the propensity for alternans and skipped beats. Notably, the effect of BPA-treatment on calcium handling was partially reversible. Our data suggest that acute BPA exposure could precipitate secondary adverse effects on contractile performance and/or electrical alternans, both of which are dependent on intracellular calcium homeostasis.

## Introduction

Bisphenol-a (BPA) is a high volume production chemical – with more than 8 million pounds produced worldwide each year. BPA is commonly used in the manufacturing of polycarbonate plastics, polyvinyl chloride plastics, resins, and thermal printing applications^1^. Humans are unintentionally exposed to bisphenols through contact with consumer products and/or medical devices, which leach BPA under normal conditions of use. Consequently, widespread and continuous exposure to bisphenols can occur through dietary intake, inhalation, dermal or intravenous exposure. Indeed, biomonitoring studies suggest that >90% of the general population is exposed to detectable levels of BPA through environmental exposure^1,2^. In comparison, intensive care patients are often exposed to extraordinarily high BPA concentrations due to medical procedures that employ BPA-containing plastic products (i.e., nasogastric tube, cardiopulmonary bypass or extracorporeal membrane oxygenation circuits, intravenous tubing, catheters)^3–5^. Once inside the body, BPA is biologically active – exerting widespread effects through endocrine disruption, genomic and non-genomic mechanisms^6–8^.

Recent studies have shown that BPA exposure negatively impacts cardiac electrophysiology and excitation-contraction coupling, using adult rodent models^9–12^. Our laboratory previously reported a linear dose-response relationship between acute BPA exposure (15 min) and impaired electrical and mechanical function, using excised Langendorff-perfused hearts from adult Sprague-Dawley rats^9,10^. Specifically, increasing BPA concentrations resulted in prolonged atrioventricular conduction time, slowed epicardial conduction velocity, decreased left ventricular developed pressure and reduced cardiac contractility (10^−9^–10^−4^M)^9,10^. Similarly, Pant *et al*. showed that increasing concentrations of BPA causes a negative inotropic and chronotropic effect on adult atrial preparations^11^. BPA exposure has also been linked to an increase in the incidence of arrhythmias and spontaneous aftercontractions in isolated adult cardiomyocytes, which may be attributed to calcium leak from the sarcoplasmic reticulum (SR)^12^. The effects of BPA on individual processes of calcium handling (i.e., L-type calcium current, SR calcium uptake, calcium spark frequency) were previously shown to have a monotonic dose response relationship in adult cells^13^.

Epidemiological studies have also shown a correlation between BPA exposure and cardiovascular pathologies, including an increased risk of hypertension, angina, myocardial infarction and reduced heart rate variability^14–23^. These associations are worrisome to the general public – but even more concerning for vulnerable populations who are exposed to exceedingly high BPA concentrations (i.e., intensive care patients, industrial workers)^3–5,24–26^ or those who are developmentally susceptible to chemical exposures (i.e., developing fetus, children)^27^. Indeed, young children with an underdeveloped metabolic system can be exposed to chemical contaminants for longer periods of time, due to slowed chemical processing and elimination. As examples, biomonitoring studies have reported maximal BPA concentrations of 0.1–0.4 × 10^−5^M in neonatal intensive care patients^3,4^. Taken together, neonatal and pediatric intensive care patients are at the greatest risk for bisphenol exposure – yet, the impact of BPA on pediatric cardiac function is unknown.

Previous studies have highlighted the link between BPA and altered cardiac functionality; although, to date, these studies have been limited to adult models. Yet, significant developmental differences exist between the immature and adult heart, including: ion channel expression and localization, development of the t-tubule system, and maturation of the excitation-contraction machinery. Developmental differences in the calcium-handling machinery of pediatric hearts could precipitate an exaggerated response to BPA chemical exposure. Based on our previously published data^9,10^, we sought to further examine the linear dose response relationship between BPA and impaired cardiac function. Specifically, we investigated the sensitivity of neonatal cardiomyocytes to BPA exposure by monitoring automaticity, excitability, intracellular calcium handling – and examined the reversibility of such effects.

## Results

### Acute BPA exposure does not negatively influence neonatal cardiomyocyte viability

**Figure 1:**
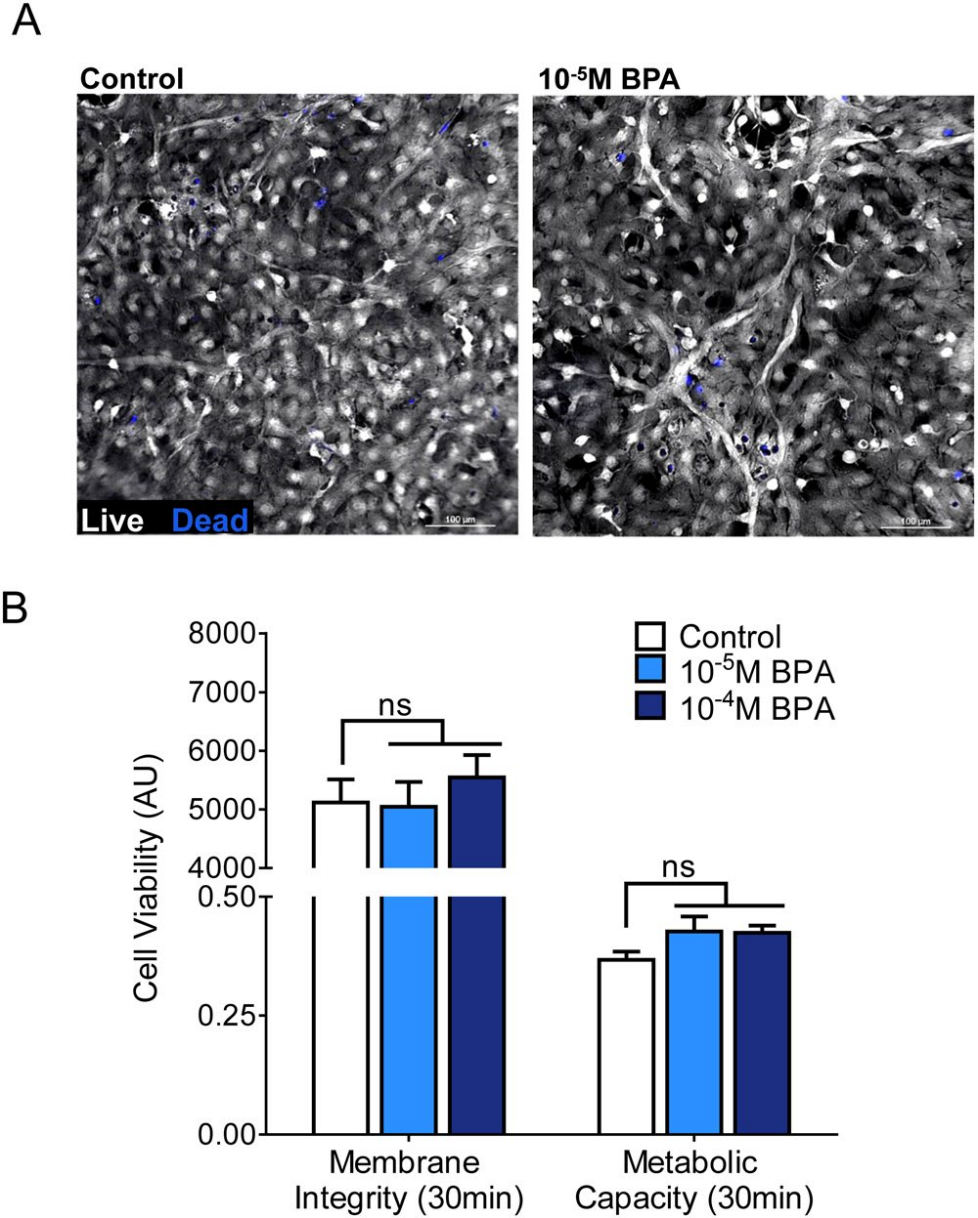
Acute BPA exposure does not impair cardiomyocyte viability. Cardiac cell viability was assessed following 30-min exposure to vehicle control or increasing concentrations of BPA. (**A**) Confluent monolayer of neonatal cardiomyocytes labeled with calcein-AM (white) and ethidium homodimer-1 (blue) to assess cell viability via membrane integrity (100 μm scale). (**B**) Cell viability measured via membrane integrity (left, as described above) and via metabolic capacity (right, resazurin-based assay). au = arbitrary units, ns = not significantly different, *p ≤ 0.05, n = 4.

Patient contact with BPA-containing medical products can result in urinary concentrations that reach micromolar levels^3–5,28,29^. *In vitro* exposure to similar concentrations has been shown to induce cytotoxicity in multiple cell types, including pancreatic islet cells, monocytes and hepatocytes^30–32^. Such an impact on cardiomyocyte viability would also effect cell structure, metabolism and/or intracellular ATP production – and impede basal cardiomyocyte functionality. With this in mind, the effect of BPA treatment on cardiomyocyte viability was assessed prior to performing secondary phenotypic testing. Cardiac cells were loaded with either a metabolic indicator dye or dual-labeled to measure cell membrane integrity (Fig. 1A). No significant difference in cell viability was observed between control and BPA-supplemented cardiomyocytes (30 min) using either assay, even at maximal concentrations (10^−5^–10^−4^M, Fig. 1B).

### Acute BPA exposure reduces cardiomyocyte automaticity and excitability

**Figure 2:**
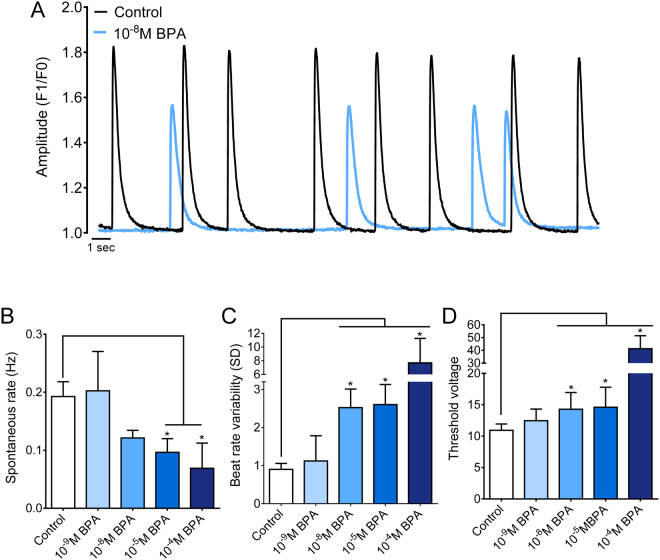
Acute BPA exposure decreases the spontaneous beating rate and cardiac excitability, and increases beat rate variability. Cardiomyocyte spontaneous beating rate was monitored following 15-min exposure to either vehicle control or BPA-supplemented media. (**A**) Representative example traces of neonatal cardiomyocytes under control conditions, or media supplemented with 10^−8^M BPA. (**B,C**) Mean spontaneous beating frequency (Hz) and beat rate variability measurements (SD of interbeat interval). (**D**) Minimum threshold voltage required for excitation via field stimulation. AUF = arbitrary units of fluorescence, F1 = peak fluorescence amplitude, F0 = baseline fluorescence. *p ≤ 0.05, n ≥ 4.

Confluent neonatal cardiomyocyte monolayers undergo phase synchronization and exhibit coordinated spontaneous beating or automaticity in culture. The intrinsic spontaneous beating rate (SBR) of cardiomyocytes is determined by a balance between inward and outward currents^33–35^ and calcium oscillations (e.g., “calcium clock”)^36^. Therefore, alterations in SBR can serve as a sensitive, yet cumulative index, of cardiac health and excitability. Previous studies have shown a causal relationship between BPA dose and slowed SBR, in adult rat atrial preparations^11^ and isolated adult rat whole hearts^10^. Acute BPA exposure (15 min) reduced SBR in neonatal cardiomyocyte monolayers, albeit only at high concentrations (Fig. 2B). The intrinsic SBR decreased by 50.0% in 10^−5^M and 64.3% in 10^−4^M BPA-treated samples, compared with control. We also observed a decrease in cardiomyocyte excitability in the presence of BPA (Fig. 2D). The threshold voltage required to externally pace cardiac cells increased to 14.3 ± 2.3 V in 10^−8^M, 14.6 ± 3.2 V in 10^−5^M, and 41.3 ± 10.2 in 10^−4^M BPA-treated samples compared with 10.9 ± 0.9 V control. Such an effect on electrical excitability could be related to the antagonistic effect of BPA on voltage gated sodium currents^37,38^, modifications in calcium handling, and/or alterations in cell membrane resistance between neighboring cells.

### Acute BPA exposure increases beat rate variability

Isolated cardiomyocytes exhibit beat rate variability (BRV) that reflects intrinsic cardiac regulatory mechanisms, including ion channel current, intracellular calcium handling, and cell coupling^34,39–42^. Similar to SBR measurements, BRV has been used as a cumulative index to assess cardiac safety, which can be influenced by a variety of proteins involved in excitation-contraction coupling^43^. Greater variance in the interbeat interval is commonly observed in poorly coupled cardiac networks or in the presence of drugs that hinder calcium handling^34^. Neonatal cardiomyocyte monolayers exhibited a regular rhythmic beating pattern under control conditions (Fig. 2A). In comparison, acute BPA exposure (15 min) increased the BRV by +179% and +189% in 10^−8^M and 10^−5^M BPA-treated samples, respectively (Fig. 2A,C). BRV also increased at the highest BPA concentration tested (10^−4^M), although the mean value was influenced by an exaggerated decrease in SBR (Fig. 2B) which resulted in complete cessation of automaticity in 57% of samples (data not shown).

### Acute BPA exposure alters intracellular calcium handling

**Figure 3:**
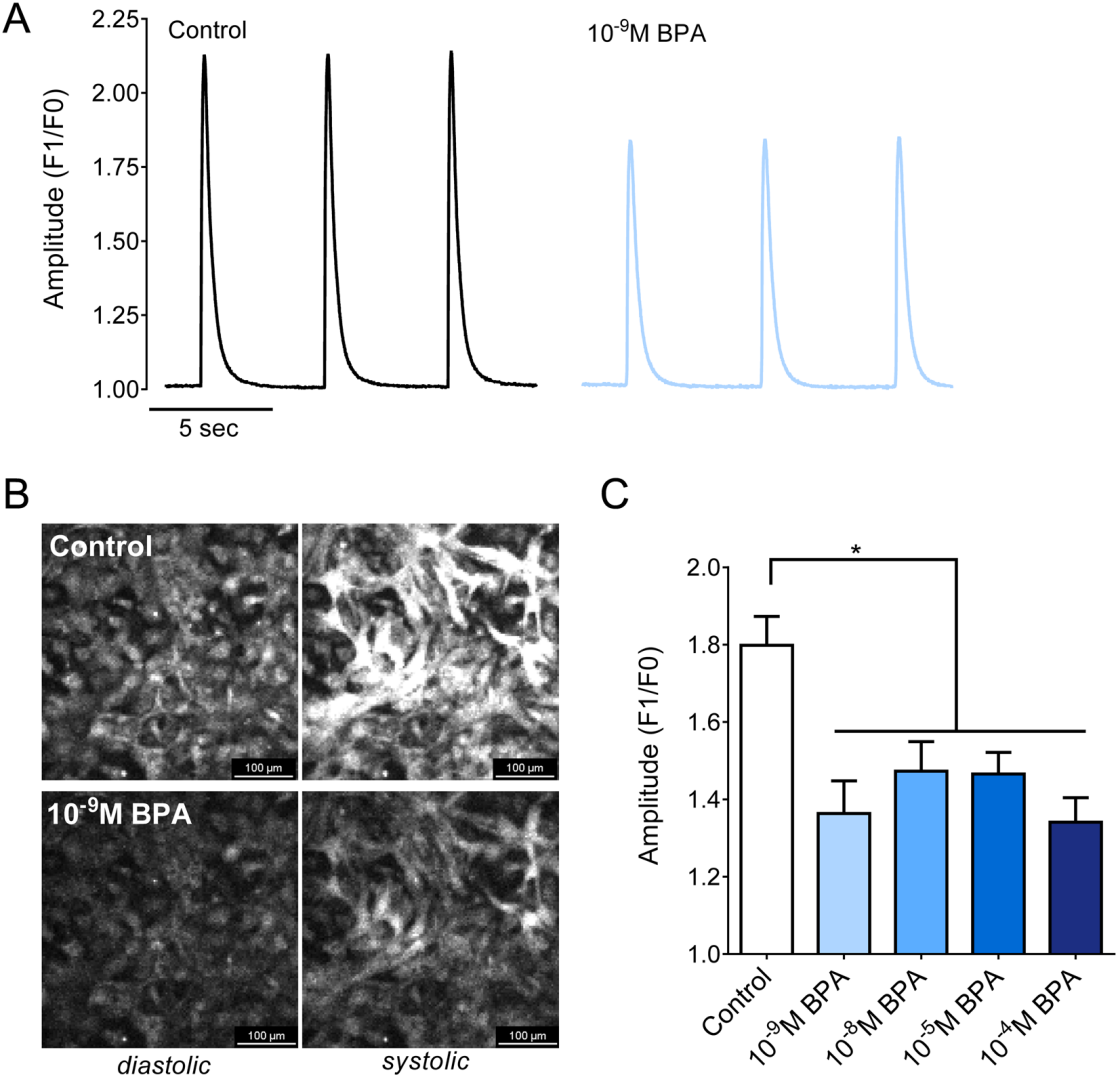
Acute BPA exposure reduces calcium transient amplitudes. (**A**) Representative CaT recorded from neonatal cardiomyocytes under control conditions, and following supplementation with 10^−8^M BPA. Cardiac monolayers were paced at 0.2 Hz using field stimulation. (**B**) Representative images show the fluorescence intensity of Fluo-4AM corresponding to diastolic (baseline) and systolic intracellular calcium (peak). (**C**) Mean CaT amplitude measured by fluorescent calcium indicator dye (Fluo4-AM), F1 = peak fluorescence amplitude, F0 = baseline fluorescence, *p ≤ 0.05, n ≥ 15.

Calcium is a vital regulator of cardiac automaticity, beat rate variability, contraction and relaxation^33,44,45^. We previously reported that BPA exposure can alter calcium handling in adult rat hearts, resulting in impaired mechanical function and decreased cardiac contractility^9^. Although, structural differences (i.e., rudimentary t-tubules and calcium release units)^46^ lead to variations in the calcium handling of immature cardiomyocytes (e.g., less pronounced calcium-induced calcium release) compared with adult cells. Calcium transients were recorded from neonatal cardiomyocytes loaded with Fluo-4AM, which exhibits an increase in fluorescence upon binding to calcium ions during systole (Fig. 3). Neonatal cardiomyocytes were externally paced, at room temperature, to negate rate dependent differences in calcium kinetics. Figure 3 shows a decrease in the peak CaT amplitude across all BPA doses tested, ranging from 18.12 to 25.45% compared with control. Such a reduction in CaT amplitude suggests a decrease in SR calcium loading, as previously described^47,48^. Importantly, the latter was not attributed to nonspecific fluorescence decay, since calcium transient amplitudes decreased by 0.9 ± 0.7% during repeated imaging under control conditions (basal vs 20 min recording).

**Figure 4:**
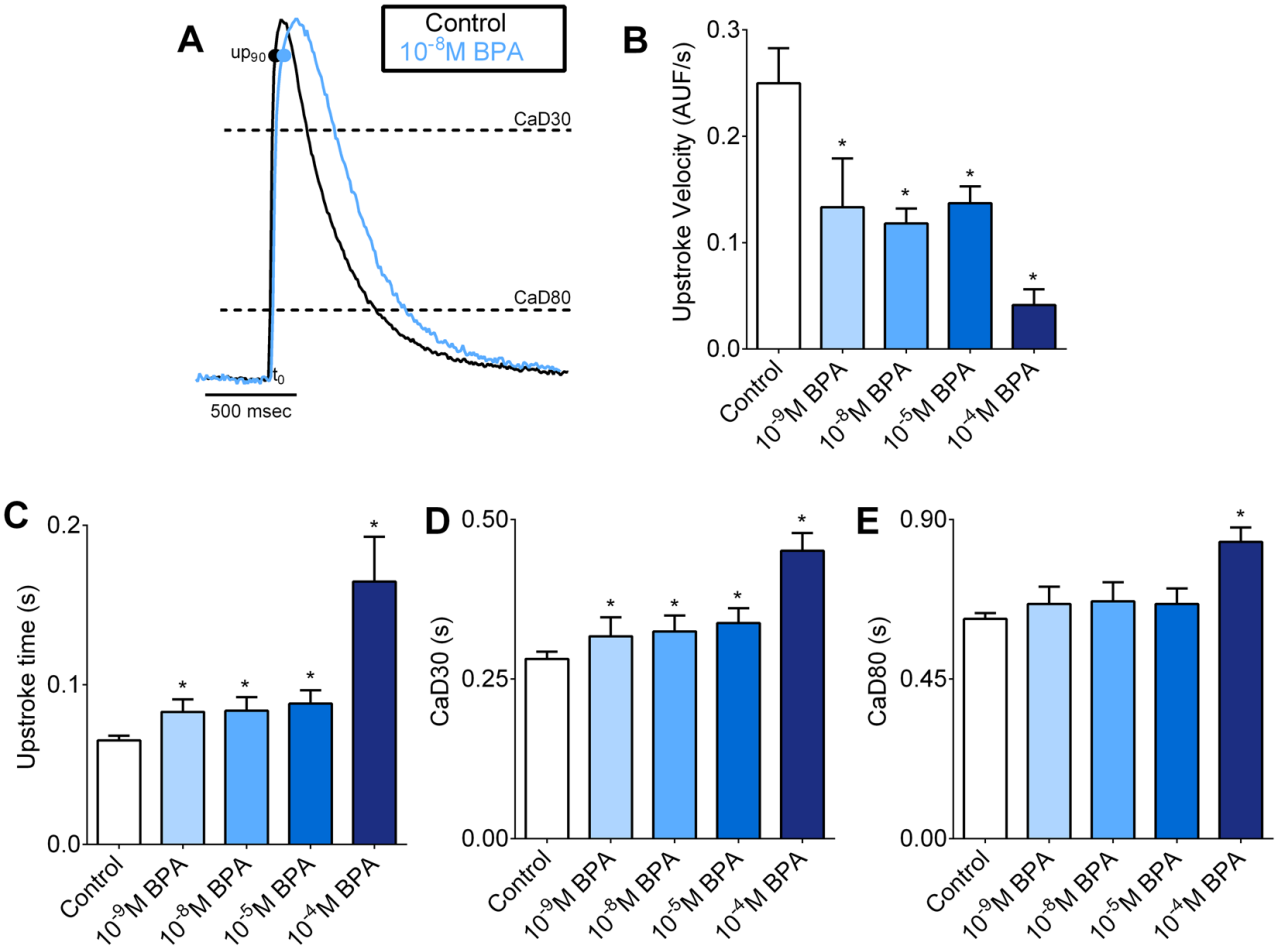
Acute BPA exposure slows intracellular calcium handling. (**A**) CaT (amplitude normalized) recorded from neonatal cardiomyocyte monolayer under control conditions, and after supplementation with 10^−8^M BPA. (**B**–**E**) CaT parameters measured under external pacing, including (**B**) upstroke velocity, (**C**) upstroke time, (**D**) 30% CaT duration (CaD30) and (**E**) 80% CaT duration (CaD80). s = seconds, ns = not significantly different, AUF = arbitrary units fluorescence, *p ≤ 0.05, n ≥ 10.

Pace-induced CaT were recorded, normalized (Fig. 4A), and temporal attributes of calcium handling were quantified. BPA-treatment slowed the time to peak (90% upstroke time), and CaT upstroke velocity beginning at low nanomolar concentrations (Fig. 4). The upstroke time increased from 65.1 ± 2.9 msec in control samples to 83.1 + 7.7 msec in 10^−9^M BPA and 164.7 + 28 msec in 10^−4^M BPA-treated samples (Fig. 4A–C). We also observed modest prolongation of CaD30 (duration from activation to 30% relaxation time) from 281.6 ± 11.1 msec in control samples to 317 ± 29.5 msec in 10^−9^M BPA and 415.2 ± 27.7 msec in 10^−4^M BPA-treated samples (Fig. 4D). Taken together, these results suggest that calcium release and reuptake to the sarcoplasmic reticulum can be hindered in BPA-treated samples.

### BPA increases the incidence of calcium transient alternans and alters restitution

**Figure 5:**
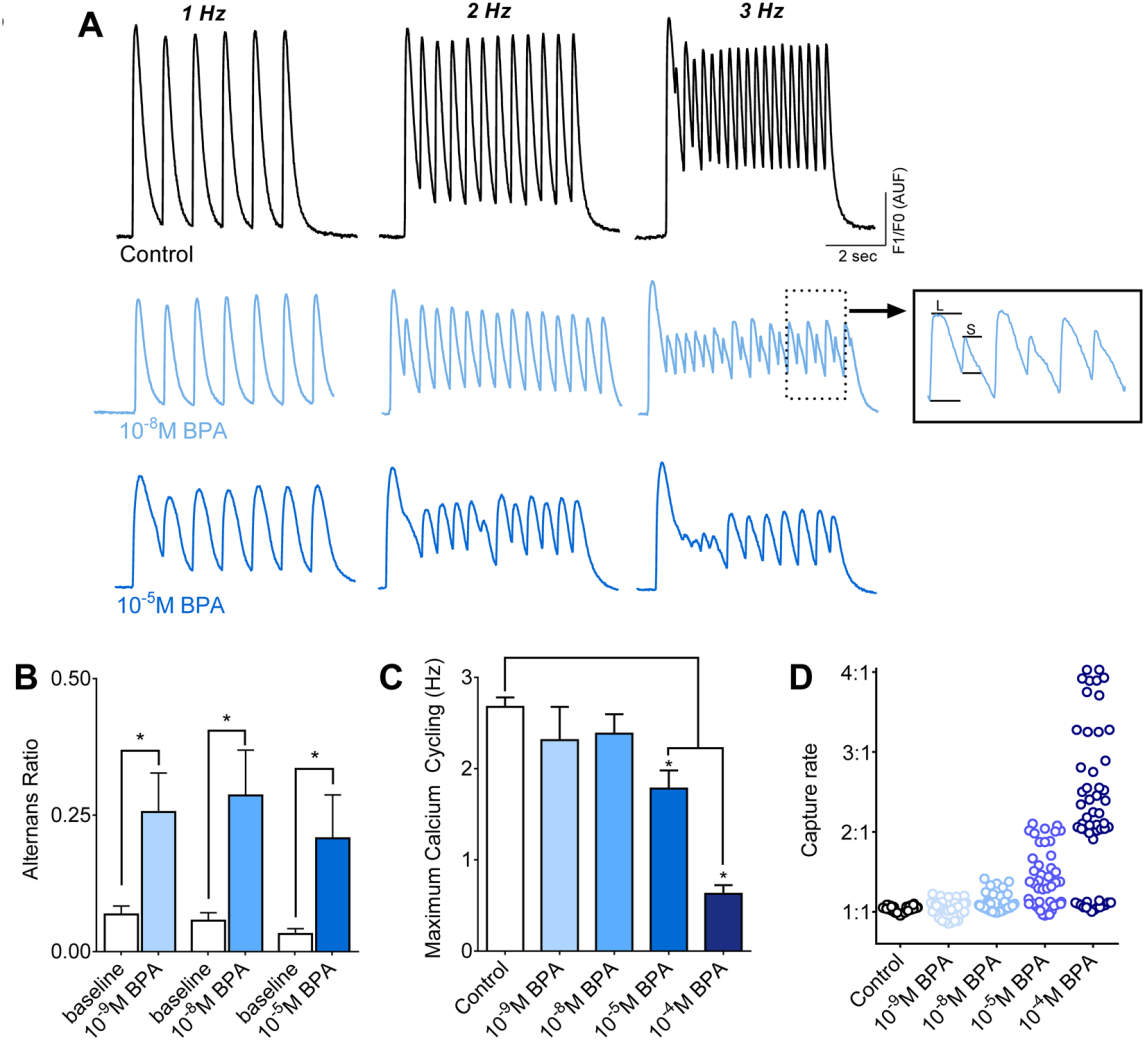
Acute BPA exposure promotes calcium transient alternans and impairs the rate of calcium cycling. (**A**) Pace-induced CaT recorded at multiple pacing frequencies (1–3 Hz) under control conditions, or following BPA-exposure. Representative example depicts CaT alternans with alternating long (L) and short (S) duration times following exposure to 10^−8^M (inset). Higher BPA concentrations (10^−5^M) slow calcium cycling, resulting in loss of capture at faster pacing rates. (**B**) Magnitude of CaT alternans expressed as a ratio [1-(CaT small/CaT large)]. (**C**) Maximum calcium cycling frequency. (**D**) Loss of 1:1 capture (2 Hz external pacing frequency). *p ≤ 0.05, n ≥ 8.

Cardiac cells display mechanical restitution, whereby a period of time is required after each contraction before another contraction of the sample amplitude can be elicited^45^. Consequently, underlying changes in cardiac refractoriness may go unnoticed at slower beating rates, but become identifiable as the beating frequency is increased. Neonatal cardiomyocytes were subjected to pacing frequencies that increased stepwise (Fig. 5A), and cell refractoriness was measured by the incidence and magnitude of CaT alternans. An alternans ratio was calculated to gauge the severity of alternans, or alternating large and small amplitudes [1-(small CaT/large CaT)]^49,50^. At the fastest pacing rate tested (3 Hz), acute BPA treatment increased the magnitude of beat-to-beat CaT alternans across all concentrations tested (Fig. 5B). Whereas substantial lengthening of refractoriness resulted in skipped beats at higher BPA concentrations (Fig. 5C,D). Acute BPA exposure decreased the maximum calcium cycling frequency to 1.8 Hz in 10^−5^M BPA and 0.6 Hz in 10^−4^M BPA, compared with 2.7 Hz in control samples (Fig. 5A,C). Cardiac refractoriness was evident at pacing rates >1 Hz, where the 1:1 capture rate decreased due to skipped or dropped beats at increasing BPA concentrations (Fig. 5D, 2 Hz pacing rate). These data suggest that BPA decreases the threshold for pacing-induced alternans and lengthens the refractoriness of calcium release. The latter may be attributed to changes in SR calcium release and/or calcium reuptake^51^, which leads to calcium instabilities.

### BPA-induced alterations in calcium handling are partially reversible

**Figure 6:**
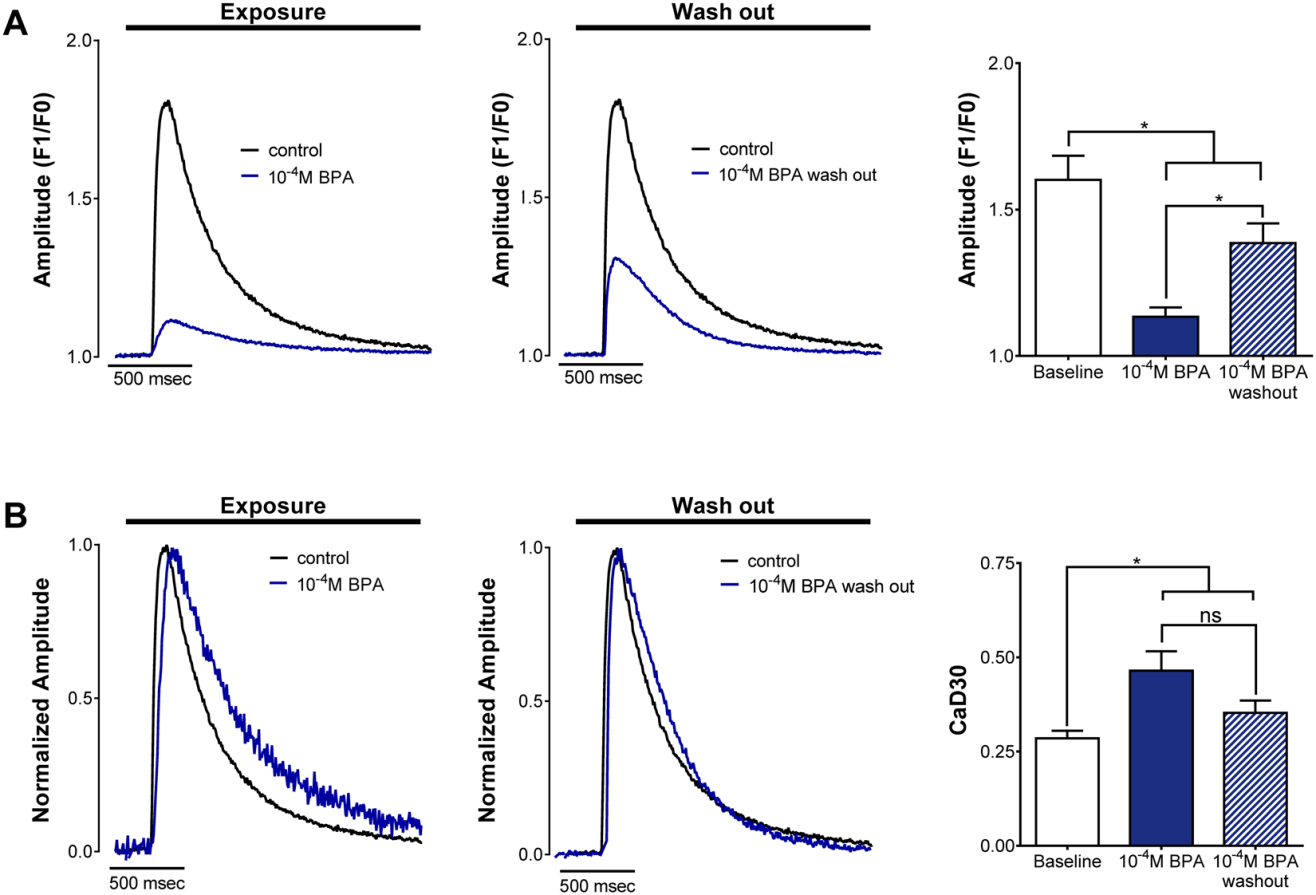
Reversibility of BPA-induced changes in calcium handling. (**A**) CaT amplitude is diminished following 15-min exposure to 10^−4^M BPA (left panel); this effect is partially reversible when 10^−4^M BPA is washed out (middle panel, right panel). (**B**) Normalized calcium signals shown prolonged CaT upstroke time and duration time following 15-min exposure to 10^−4^M BPA (left panel); CaT duration effects are partially negated following 1-hr wash out. F1 = peak fluorescence amplitude, F0 = baseline fluorescence, ns = not significantly different, *p ≤ 0.05, n ≥ 6.

Human pharmacokinetic studies suggest that BPA is rapidly metabolized, with a half-life of a few hours^52–54^. With this in mind, we aimed to determine whether the effects of BPA on calcium handling were reversible. CaT were recorded from neonatal cardiomyocytes under control conditions, following acute 15 min exposure to maximal concentrations of BPA (10^−4^M) and again after 1 hr wash out (Fig. 6). CaT amplitude decreased by −29.2% and CaT duration increased by +62.9% after exposure to 10^−4^M BPA, compared with control. Following wash out, calcium handling parameters partially recovered, but remained significantly different from baseline control recordings. CaT amplitude remained −13.4% lower (Fig. 6A) and CaT duration remained +23.8% longer (Fig. 6B) in wash out samples, compared with baseline control recordings.

## Discussion

Significant developmental differences exist between the immature and adult heart, including: ion channel expression and localization, development of the t-tubule system, and maturation of the excitation-contraction machinery. Calcium handling also matures dramatically during development – calcium oscillations first serve a pacemaker function (“calcium clock”)^36,55^, whereas later, action potential-driven calcium-induced calcium release becomes essential for synchronized muscle contraction^46^. Indeed, inefficient excitation-contraction coupling is observed in myocytes with rudimentary t-tubules and reduced coupling between L-type calcium channels and ryanodine receptors^46^. Such an immature phenotype can increase the propensity for arrhythmias, reduce calcium transient amplitude, and diminish contractile force^56^. These unique characteristics can impede the extrapolation of BPA cardiotoxicity data between adult^9,10,12,57,58^ and pediatric experimental studies.

Although pediatric populations may be more vulnerable to endocrine disrupting chemicals (“window of vulnerability”^27,59,60^) – the effect of BPA exposure on immature heart cells is unknown. To address this knowledge gap, we examined the impact of BPA exposure on excitation-contraction coupling in neonatal cardiomyocytes. The current study investigated a wide range of BPA doses that mimic environmental, clinical and supraphysiological exposure levels (10^−9^–10^−4^M). In the presented study, acute (15 min) BPA exposure slowed the SBR, increased BRV, and diminished cardiac cell excitability at nanomolar concentrations. We also detected significant, although modest, changes in calcium transient kinetics at slow pacing rates – which were coupled with an increased propensity for calcium transient alternans and prolonged refractoriness at faster pacing rates. Importantly, the observed effects on calcium handling were at least partially reversible. The presented study supports our previous finding that BPA exposure can impact cardiac electrical and mechanical function^9,10^, and highlights the importance of addressing pediatric heart vulnerability to endocrine-disrupting chemicals.

### Spontaneous beating rate, beat rate variability and excitability

Although isolated adult cardiomyocytes are quiescent, neonatal cardiomyocytes exhibit spontaneous synchronized beating that can serve as a sensitive and cumulative index of cardiac health and excitability. In the presented study, we observed slowing of the SBR and an increase in BRV following BPA-treatment of neonatal myocytes. *In vivo* measurements of heart rate variability can be influenced by extracardiac factors (i.e., autonomic, endocrine), whereas BRV in the neonatal myocyte model reflects the intrinsic properties of cardiac tissue (i.e., ion channel current, intracellular calcium handling, gap junction intercellular communication)^34,42^. Indeed, alterations in the BRV of isolated cardiomyocytes has previously been reported in poorly coupled cell networks, in the presence of drugs that hinder calcium handling, or following funny current inhibition^34,41^. Whereas slowing of the SBR may be attributed to a disturbance in spontaneous calcium oscillations (e.g., calcium leak from SR)^61^, and outward calcium/inward sodium flux via the sodium-calcium exchanger^46,62,63^. This finding concurs with published studies noting a negative chronotropic response to BPA-treatment in isolated adult atrial preparations^11^ and excised whole hearts^10^. We also observed a decrease in cell excitability following BPA-treatment, which may be related to the antagonistic effect of BPA on voltage gated sodium currents^37,38^, activation of potassium channels^64^, modifications in calcium handling, and/or alterations in cell membrane resistance between neighboring cells. Indeed, BPA-treatment has been shown to influence gap junction functionality^65–67^, although these studies have not yet been confirmed in a cardiac model.

### Calcium handling, calcium transient alternans and refractoriness

Alterations in calcium cycling will also influence excitation-contraction coupling – the process linking electrical excitation to contraction. Liang, *et al*. previously reported that BPA exposure inhibited L-type calcium channel current and modified the calcium release/reuptake kinetics of the SR in adult myocytes^13^. The authors noted an increase in the propensity for spontaneous aftercontractions in adult cardiac cells and arrhythmias in adult animals, which was attributed to calcium leak from the SR^12,68^. In the current study, we observed modest slowing of calcium cycling kinetics (upstroke, upstroke velocity, CaD30) that coincided with an increased propensity for alternans and skipped beats. Calcium transient alternans can result from an imbalance between calcium release (e.g., ryanodine receptor^69^) and reuptake (e.g. SR calcium ATPase, SERCA), and/or an increase in ryanodine receptor refractoriness^70^, which leads to calcium instabilities. Importantly, intracellular calcium alternans have been linked to action potential alternans, t-wave alternans and re-entrant arrhythmias^71–73^. Additional studies are needed to ascertain whether BPA exposure increases the propensity for electrical alternans.

In the present study, we also observed a decrease in the CaT amplitude of BPA-treated samples, indicating a reduction in the amount of calcium released from the SR with each contraction. Given the relationship between intracellular calcium concentration, myofilament sensitivity, and contractile force – diminished calcium release would explain the negative inotropic response observed in atrial and whole heart preparations^9,11^. Diminished CaT amplitudes are also observed in situations of reduced SERCA activity and/or diminished ryanodine receptor synchronization^74,75^. Given the immediate effects of BPA, post-translational modifications of key calcium handling proteins (e.g. phospholamban, ryanodine) and/or interference with ion channel current (e.g. L-type calcium channel) may explain our observations.

### Reversibility of cardiac effects

In the presented study, we showed that the effects of high-dose BPA exposure (10^−4^M; acute 15-min) on calcium handling are partially reversible. This finding coincides with previously published studies showing that the effects of BPA on ion channel functionality are likely reversible – and presents important insight for human risk assessment. Deutschmann, *et al*. showed that BPA (10^−5^–10^−4^M) is a potent blocker of voltage gated calcium channels, but noted that current inhibition was almost fully reversible in cardiac myocytes^76^. Similarly, Wang, *et al*. noted the rapid inhibitory effects of BPA on voltage-gated sodium channels in neurons; this inhibitor effect was concentration-dependent (2–12 × 10^−5^M) and mostly reversible after a short wash out period^38^. BPA treatment has also been shown to inhibit gap junction intercellular communication and the outward current of connexin hemichannels, although the reversibility of these effects may be cell or connexin-isoform specific (connexin-43 vs connexin-46)^65–67^. Finally, Asano, *et al*. reported that BPA-treatment (10^−6^–10^−4^M) reversibly activates Maxi-K channels in coronary smooth muscle cells^64^. It is unclear whether similar results translate to the human myocardium^65,66^.

### Limitations

The scope of the current study was limited to the effect of acute (15 min) BPA exposure on neonatal rat cardiomyocytes. We hypothesize that the quick, partially-reversible action of BPA is likely due to ion channel interactions, as described in other cell types, and/or post-translational modifications of key calcium-handling proteins. Additional studies are needed to fully interrogate the underlying mechanisms of bisphenol chemicals, which are likely multifactorial and may differ entirely in instances of longer-term exposure. Our previous findings reported a linear dose-response relationship between BPA and whole heart physiology; therefore, our current study included a range of BPA concentrations that covered environmental (10^−9^–10^−8^M), maximal clinical (10^−5^M) and supraphysiological exposure levels (10^−4^M). The latter allowed our group to show that phenotypic changes were not solely attributed to cardiomyocyte death and were partially reversible, even at extraordinarily high concentrations. Nevertheless, we cannot rule out alternative cardiac outcomes at different exposure levels, since BPA may exert a non-monotonic dose-response^77,78^. Finally, the current study was limited to neonatal cell preparations from newborn rats (1 day old, male and female mixed litter), which prevented the examination of sex-specific cardiac effects in response to BPA exposure. Since BPA is estrogenic^12^, additional studies are warranted to determine whether the observed effects on cardiac physiology are exacerbated in females.

## Conclusion

Plastics have revolutionized medical device technology, transformed hematological care and transfusion medicine, and facilitated modern cardiology procedures. Despite these benefits, the ubiquitous nature of plastics has also raised concerns pertaining to human health risks – particularly in sensitive pediatric populations. For the first time, we have shown that a commonly used plastic chemical (BPA) can have an immediate effect on neonatal cardiomyocyte beating rate, intracellular calcium handling, and the incidence of alternans. Our data suggests that incidental BPA exposure may precipitate secondary adverse effects on contractile performance and/or electrical alternans, both of which are dependent on intracellular calcium homeostasis. Additional studies are necessary to determine the effects of bisphenol exposure on pediatric whole heart physiology and the impact of prolonged exposure, which may more closely mimic clinical exposure. Given the elevated risk of BPA exposure in intensive care settings, our data highlights the importance of incentivizing the development, manufacturing, and clinical adoption of alternative biomaterials to improve patient safety.

## Materials and Methods

### Animals

Animal protocols were approved by the Institutional Animal Care and Use Committee of the Children’s Research Institute, and followed the National Institutes of Health’s *Guide for the Care and Use of Laboratory Animals*. Neonatal rat cardiomyocytes were isolated from 1 day old Sprague-Dawley rats (male and female, mixed litter) by an enzymatic digestion protocol, as previously described^79,80^. Cells were plated on laminin-coated coverglass (10^5^ cells/cm^2^), and maintained under standard cell culture conditions in Dulbecco-modified minimum essential medium supplemented with 5% Fetal bovine serum, 10 U/ml penicillin, and 1 μg/ml streptomycin (Thermo Fisher Scientific). Three days after plating, neonatal rat cardiomyocytes formed a confluent monolayer and exhibited strong synchronized contractions. Cardiac cells were used thereafter for subsequent experiments, as previously described^48,80,81^.

### Experimental Protocol

An analytical standard of bisphenol-a (≥99% purity, Sigma Aldrich) was dissolved in 100% ethanol to provide a 500 mM stock solution. The stock solution was then diluted in Tyrode salt solution (Sigma Aldrich) to obtain final working concentrations of 10^−9^–10^−4^M BPA. The highest possible ethanol concentration was used as vehicle control (0.001%). Cardiomyocytes were incubated with BPA-supplemented media for 15 minutes for all live-cell recordings.

### Cell viability measurements

Confluent adherent cardiomyocytes were exposed to maximum BPA concentrations (10^−5^–10^−4^M) for 30 min and cell viability was quantified. A live/dead viability dye-based assay (Thermo Fisher Scientific) was used to visualize and quantify cell membrane integrity in live (calcein-AM, 495 nm excitation/517 nm emission) versus dead cells (ethidium homodimer-1, 517 nm excitation/617 nm emission). Widefield fluorescent images were acquired from (4) different fields of view, per coverslip, using a Nikon TiE microscope system equipped with a scMOS camera (Andor Zyla 4.2 plus) and deconvolution software (Nikon Elements, NVIDIA GPU). Relative viability was measured by the total cell area corresponding to the live (calcein-AM) or dead (ethidium homodimer-1) labeling, which was quantitated by fluorescent histogram profiles. In a second set of experiments, a resazurin assay was used to measure cell viability based on metabolic capacity (PrestoBlue, Thermo Fisher Scientific). Relative viability was measured as the difference between absorbance at 600 nm and 570 nm, which corresponds to conversion of resazurin to resorufin by live metabolically active cells.

### Live imaging of calcium transients (CaT)

Confluent layers of cardiomyocytes were loaded for 40 min at room temperature with 10 μM Fluo-4AM (Thermo Fisher Scientific), a fluorescent calcium indicator, and then washed in dye-free Tyrode salt solution^47,80^. Cell monolayers were then exposed to vehicle control or BPA-supplemented Tyrode media for 15 min at room temperature. Spontaneous (37 °C) and pace-induced calcium transients (CaT) were acquired using a Nikon TiE microscope system (23 °C), equipped with 470 nm excitation LED (SpectraX, Lumencor), 505–530 nm emission filter, and Andor iXon 860 EMCCD camera (~170 fps, 128 × 128). Cardiac monolayers were paced using field stimulation (monophasic, 5 msec pulses, 1.5 × threshold voltage, 0.2–3 Hz frequency; Grass Technologies).

### Analysis of beat rate variability and calcium kinetics

The following parameters were determined from raw CaT signals: amplitude (F_1_/F_0_), upstroke time (duration from activation to 90% of maximum amplitude), upstroke velocity (Δfluorescence/s), 30% and 80% CaT duration time (duration from activation to 30% or 80% reuptake time)^47,48^. The beginning of the CaT upstroke was defined by the initial deflection from baseline (max d^2F^/dt^2^). Calcium alternans were induced by increasing the pacing frequency, stepwise from 0.2 Hz to 3 Hz. The calcium alternan ratio was calculated as [1-(CaT small/CaT large)], where CaT small and CaT large are the corresponding fluorescence amplitudes from a pair of alternating calcium transients^49,50^. Resultant values range from 0 (no alternans) to 1 (maximum alternans). Beat rate variability (BRV) was calculated as the standard deviation of the interbeat interval.

### Statistical Analysis

All values are expressed as mean + SEM, with p < 0.05 considered statistically significant, denoted with an asterisk (*). Lowest observed adverse effect level was determined within a treatment group using ANOVA followed by posthoc multiple comparisons testing (individual coverslips) or multiple paired Student t-tests (two-tailed; baseline control vs treated), as previously described (Prism, Graphpad Software Inc)^9,82^. Representative traces and images are shown.

## References

[1] Vandenberg LN (2012). Urinary, circulating, and tissue biomonitoring studies indicate widespread exposure to bisphenol A. Cien. Saude Colet..

[2] Calafat AM, Ye X, Wong LY, Reidy JA, Needham LL (2008). Exposure of the U.S. population to bisphenol A and 4-tertiary-octylphenol: 2003-2004. Environ. Health Perspect..

[3] Calafat AM (2009). Exposure to bisphenol A and other phenols in neonatal intensive care unit premature infants. Environ. Health Perspect..

[4] Duty SM (2013). Potential sources of bisphenol A in the neonatal intensive care unit. Pediatrics.

[5] Huygh J (2015). Considerable exposure to the endocrine disrupting chemicals phthalates and bisphenol-A in intensive care unit (ICU) patients. Environ. Int..

[6] Li L (2015). The Molecular Mechanism of Bisphenol A (BPA) as an Endocrine Disruptor by Interacting with Nuclear Receptors: Insights from Molecular Dynamics (MD) Simulations. PLoS One.

[7] Welshons WV, Nagel SC, vom Saal FS (2006). Large Effects from Small Exposures. III. Endocrine Mechanisms Mediating Effects of Bisphenol A at Levels of Human Exposure. Endocrinology.

[8] Posnack NG (2014). The Adverse Cardiac Effects of Di(2-ethylhexyl)phthalate and Bisphenol A. Cardiovasc Toxicol.

[9] Posnack NG (2015). Physiological response of cardiac tissue to Bisphenol A: alterations in ventricular pressure and contractility. Am. J. Physiol. Heart Circ. Physiol..

[10] Posnack NG (2014). Bisphenol A exposure and cardiac electrical conduction in excised rat hearts. Environ. Health Perspect..

[11] Pant J, Ranjan P, Deshpande SB (2011). Bisphenol A decreases atrial contractility involving NO-dependent G-cyclase signaling pathway. J. Appl. Toxicol..

[12] Yan S (2011). Bisphenol A and 17beta-estradiol promote arrhythmia in the female heart via alteration of calcium handling. PLoS One.

[13] Liang Q, Gao X, Chen Y, Hong K, Wang H-S (2014). Cellular mechanism of the nonmonotonic dose response of bisphenol A in rat cardiac myocytes. Environ. Health Perspect..

[14] Aekplakorn W, Chailurkit L-O, Ongphiphadhanakul B (2015). Association of serum bisphenol a with hypertension in thai population. Int. J. Hypertens..

[15] Bae S, Hong Y-C (2015). Exposure to bisphenol A from drinking canned beverages increases blood pressure: randomized crossover trial. Hypertension.

[16] Bae S, Kim JH, Lim Y-H, Park HY, Hong Y-C (2012). Associations of bisphenol A exposure with heart rate variability and blood pressure. Hypertension.

[17] Shankar A, Teppala S (2012). Urinary bisphenol A and hypertension in a multiethnic sample of US adults. J. Environ. Public Health.

[18] Lang IA (2008). Association of urinary bisphenol A concentration with medical disorders and laboratory abnormalities in adults. JAMA.

[19] Lind PM, Lind L (2011). Circulating levels of bisphenol A and phthalates are related to carotid atherosclerosis in the elderly. Atherosclerosis.

[20] Melzer D (2012). Urinary bisphenol a concentration and angiography-defined coronary artery stenosis. PLoS One.

[21] Melzer D (2012). Urinary bisphenol A concentration and risk of future coronary artery disease in apparently healthy men and women. Circulation.

[22] Melzer D, Rice NE, Lewis C, Henley WE, Galloway TS (2010). Association of urinary bisphenol a concentration with heart disease: evidence from NHANES 2003/06. PLoS One.

[23] Shankar, A., Teppala, S. & Sabanayagam, C. Bisphenol A and Peripheral Arterial Disease: Results from the NHANES. *Environ. Health Perspect*, 10.1289/ehp.1104114 (2012). 10.1289/ehp.1104114 PMC3440106 22645278

[24] Wang, F. *et al*. High urinary bisphenol A concentrations in workers and possible laboratory abnormalities. *Occup. Environ. Med*, 10.1136/oemed-2011-100529 (2012). 10.1136/oemed-2011-100529 22562051

[25] Hines CJ (2017). Urinary Bisphenol A (BPA) Concentrations among Workers in Industries that Manufacture and Use BPA in the USA. Ann. Work Expo. Heal..

[26] Ribeiro E, Ladeira C, Viegas S (2017). Occupational Exposure to Bisphenol A (BPA): A Reality That Still Needs to Be Unveiled. Toxics.

[27] Braun JM, Hauser R (2011). Bisphenol A and children’s health. Curr. Opin. Pediatr..

[28] Braun JM (2011). Variability and predictors of urinary bisphenol A concentrations during pregnancy. Environ. Health Perspect..

[29] Sathyanarayana S, Braun JM, Yolton K, Liddy S, Lanphear BP (2011). Case report: high prenatal bisphenol a exposure and infant neonatal neurobehavior. Environ. Health Perspect..

[30] Carchia E (2015). Evaluation of low doses BPA-induced perturbation of glycemia by toxicogenomics points to a primary role of pancreatic islets and to the mechanism of toxicity. Cell Death Dis..

[31] Neri M (2015). *In vitro* Cytotoxicity of Bisphenol A in Monocytes Cell Line. Blood Purif..

[32] Nakagawa Y, Tayama S (2000). Metabolism and cytotoxicity of bisphenol A and other bisphenols in isolated rat hepatocytes. Arch. Toxicol..

[33] Sirenko O (2013). Assessment of beating parameters in human induced pluripotent stem cells enables quantitative *in vitro* screening for cardiotoxicity. Toxicol. Appl. Pharmacol..

[34] Ben-Ari M (2014). From beat rate variability in induced pluripotent stem cell-derived pacemaker cells to heart rate variability in human subjects. Hear. Rhythm.

[35] Mandel Y (2012). Human embryonic and induced pluripotent stem cell-derived cardiomyocytes exhibit beat rate variability and power-law behavior. Circulation.

[36] Lakatta EG, Maltsev VA, Vinogradova TM (2010). A coupled SYSTEM of intracellular Ca2+ clocks and surface membrane voltage clocks controls the timekeeping mechanism of the heart’s pacemaker. Circ. Res..

[37] O’Reilly AO (2012). Bisphenol a binds to the local anesthetic receptor site to block the human cardiac sodium channel. PLoS One.

[38] Wang Q (2011). Inhibition of voltage-gated sodium channels by bisphenol A in mouse dorsal root ganglion neurons. Brain Res..

[39] Billman GE (2012). Does the ‘coupled clock’ make the heart tick?. Cardiovasc. Res..

[40] Rosen MR, Nargeot J, Salama G (2012). The case for the funny current and the calcium clock. Hear. Rhythm.

[41] Zaniboni M, Cacciani F, Lux RL (2014). Beat-to-Beat Cycle Length Variability of Spontaneously Beating Guinea Pig Sinoatrial Cells: Relative Contributions of the Membrane and Calcium Clocks. PLoS One.

[42] Kucera JP, Heuschkel MO, Renaud P, Rohr S (2000). Power-law behavior of beat-rate variability in monolayer cultures of neonatal rat ventricular myocytes. Circ. Res..

[43] Abassi YA (2012). Dynamic monitoring of beating periodicity of stem cell-derived cardiomyocytes as a predictive tool for preclinical safety assessment. Br. J. Pharmacol..

[44] Bers DM (2002). Calcium and cardiac rhythms: physiological and pathophysiological. Circ Res.

[45] Bers, D. M. *Excitation-contraction coupling and cardiac contractile force*. (Springer, 2001).

[46] Louch WE, Koivumäki JT, Tavi P (2015). Calcium signalling in developing cardiomyocytes: implications for model systems and disease. J. Physiol..

[47] Posnack NG (2014). Exposure to phthalates affects calcium handling and intercellular connectivity of human stem cell-derived cardiomyocytes. PLoS One.

[48] Jaimes R (2016). Functional response of the isolated, perfused normoxic heart to pyruvate dehydrogenase activation by dichloroacetate and pyruvate. Pflügers Arch. - Eur. J. Physiol..

[49] Florea, S. M. & Blatter, L. A. Regulation of cardiac alternans by β-adrenergic signaling pathways. *Am. J. Physiol. - Hear. Circ. Physiol*. **303** (2012). 10.1152/ajpheart.00384.2012 PMC3469647 22904161

[50] Kanaporis G, Blatter LA (2015). The mechanisms of calcium cycling and action potential dynamics in cardiac alternans. Circ. Res..

[51] Shkryl VM, Maxwell JT, Domeier TL, Blatter LA (2012). Refractoriness of sarcoplasmic reticulum Ca2+ release determines Ca2+ alternans in atrial myocytes. Am. J. Physiol. Heart Circ. Physiol..

[52] Vandenberg LN, Hauser R, Marcus M, Olea N, Welshons WV (2007). Human exposure to bisphenol A (BPA). Reprod. Toxicol..

[53] Teeguarden JG, Waechter JM, Clewell HJ, Covington TR, Barton HA (2005). Evaluation of oral and intravenous route pharmacokinetics, plasma protein binding, and uterine tissue dose metrics of bisphenol A: a physiologically based pharmacokinetic approach. Toxicol. Sci..

[54] Volkel W, Colnot T, Csanady GA, Filser JG, Dekant W (2002). Metabolism and kinetics of bisphenol a in humans at low doses following oral administration. Chem. Res. Toxicol..

[55] Lakatta EG, DiFrancesco D (2009). What keeps us ticking: a funny current, a calcium clock, or both?. J. Mol. Cell. Cardiol..

[56] Fearnley CJ, Roderick HL, Bootman MD (2011). Calcium signaling in cardiac myocytes. Cold Spring Harb. Perspect. Biol..

[57] Patel BB, Raad M, Sebag IA, Chalifour LE (2013). Lifelong exposure to bisphenol a alters cardiac structure/function, protein expression, and DNA methylation in adult mice. Toxicol. Sci..

[58] Gear R, Kendziorski JA, Belcher SM (2017). Effects of bisphenol A on incidence and severity of cardiac lesions in the NCTR-Sprague-Dawley rat: A CLARITY-BPA study. Toxicol. Lett..

[59] Blesson CS, Yallampalli C (2015). Pregnancy is a new window of susceptibility for bisphenol a exposure. Endocrinology.

[60] Stacy SL (2017). Early life bisphenol A exposure and neurobehavior at 8 years of age: Identifying windows of heightened vulnerability. Environ. Int..

[61] Gambardella, J., Trimarco, B., Iaccarino, G. & Santulli, G. In*Advances in experimental medicine and biology*, 10.1007/5584_2017_106 (2017). 10.1007/978-3-319-55330-6_26 PMC6691902 28551804

[62] Zhang X-H (2015). Regionally diverse mitochondrial calcium signaling regulates spontaneous pacing in developing cardiomyocytes. Cell Calcium.

[63] Morad M, Zhang X (2017). Mechanisms of spontaneous pacing: sinoatrial nodal cells, neonatal cardiomyocytes, and human stem cell derived cardiomyocytes. Can. J. Physiol. Pharmacol..

[64] Asano S, Tune JD, Dick GM (2010). Bisphenol A activates Maxi-K (K(Ca)1.1) channels in coronary smooth muscle. Br. J. Pharmacol..

[65] Lee IK, Rhee SK (2007). Inhibitory effect of bisphenol A on gap junctional intercellular communication in an epithelial cell line of rat mammary tissue. Arch. Pharm. Res..

[66] Oh S (2015). Bisphenol A and 4-tert-Octylphenol Inhibit Cx46 Hemichannel Currents. Korean J. Physiol. Pharmacol..

[67] Li MWM, Mruk DD, Lee WM, Cheng CY (2009). Disruption of the blood-testis barrier integrity by bisphenol A *in vitro*: Is this a suitable model for studying blood-testis barrier dynamics?. Int. J. Biochem. Cell Biol..

[68] Gao X, Liang Q, Chen Y, Wang H-S (2013). Molecular mechanisms underlying the rapid arrhythmogenic action of bisphenol A in female rat hearts. Endocrinology.

[69] Santulli G, Lewis D, des G A, Marks AR, Frank J (2018). Ryanodine Receptor Structure and Function in Health and Disease. Subcell. Biochem..

[70] Wang L (2014). Optical mapping of sarcoplasmic reticulum Ca2+ in the intact heart: ryanodine receptor refractoriness during alternans and fibrillation. Circ. Res..

[71] Clusin, W. T. Mechanisms of calcium transient and action potential alternans in cardiac cells and tissues. *Am. J. Physiol. - Hear. Circ. Physiol*. **294** (2008). 10.1152/ajpheart.00802.2007 17951365

[72] Wilson LD, Rosenbaum DS (2007). Mechanisms of arrythmogenic cardiac alternans. Europace.

[73] Escobar AL, Valdivia HH (2014). Cardiac alternans and ventricular fibrillation: a bad case of ryanodine receptors reneging on their duty. Circ. Res..

[74] Niggli E (2011). Ryanodine receptors: waking up from refractoriness. Cardiovasc. Res..

[75] Zhong X (2016). Suppression of ryanodine receptor function prolongs Ca2+ release refractoriness and promotes cardiac alternans in intact hearts. Biochem. J..

[76] Deutschmann A, Hans M, Meyer R, Häberlein H, Swandulla D (2013). Bisphenol A inhibits voltage-activated Ca(2+) channels *in vitro*: mechanisms and structural requirements. Mol. Pharmacol..

[77] Vandenberg LN (2014). Non-monotonic dose responses in studies of endocrine disrupting chemicals: bisphenol a as a case study. Dose. Response..

[78] Lagarde F (2015). Non-monotonic dose-response relationships and endocrine disruptors: a qualitative method of assessment. Environ. Health.

[79] Arutunyan A, Webster DR, Swift LM, Sarvazyan N (2001). Localized injury in cardiomyocyte network: a new experimental model of ischemia-reperfusion arrhythmias. Am J Physiol Hear. Circ Physiol.

[80] Gillum N (2009). Clinically relevant concentrations of di (2-ethylhexyl) phthalate (DEHP) uncouple cardiac syncytium. Toxicol. Appl. Pharmacol..

[81] Posnack, N. G. *et al*. Exposure to phthalates affects calcium handling and intercellular connectivity of human stem cell-derived cardiomyocytes. *PLoS One* **10** (2015). 10.1371/journal.pone.0121927 PMC4370601 25799571

[82] Bokkers BG, Slob W (2005). A comparison of ratio distributions based on the NOAEL and the benchmark approach for subchronic-to-chronic extrapolation. Toxicol. Sci..

